# Dysregulation of amino acids and lipids metabolism in schizophrenia with violence

**DOI:** 10.1186/s12888-020-02499-y

**Published:** 2020-03-04

**Authors:** Xiacan Chen, Jiajun Xu, Jing Tang, Xinhua Dai, Haolan Huang, Ruochen Cao, Junmei Hu

**Affiliations:** 1 Institute of Forensic Medicine, West China School of Basic Medical Sciences & Forensic Medicine, Sichuan University, Chengdu, China; 2 Mental Health Center, West China Hospital, Sichuan University, Chengdu, China; 3 Chengdu Compulsory Medical Center, Chengdu, China; 4 West China School of Basic Medical Sciences & Forensic Medicine, Sichuan University, Chengdu, 610041 China

**Keywords:** Schizophrenia, Violence, Metabolomics, Biomarker

## Abstract

**Background:**

Many studies have related biochemical characteristics to violence and have reported schizophrenia could elevated the risk of violent behaviour. However, the metabolic characteristics of schizophrenia patients with violence (V.SC) are unclear.

**Methods:**

To explore the metabolic characteristics of schizophrenia with violence and to identify potential biomarkers, untargeted metabolomics was performed by using gas chromatography time-of-flight mass spectrometry to analyse the plasma metabolites of fifty-three V.SC and twenty-four schizophrenia patients without violence (NV.SC). Multivariate and univariate analyses were performed to identify differential metabolites and biomarkers. Violence was assessed by the MacArthur Violence Assessment Study method. Psychiatric symptoms were assessed by the Positive and Negative Syndrome Scale.

**Results:**

Multivariate analysis was unable to distinguish V.SC from NV.SC. Glycerolipid metabolism and phenylalanine, tyrosine and tryptophan biosynthesis were the differential metabolic pathways between V.SC and NV.SC. We confirmed ten metabolites and five metabolites as metabolic biomarkers of V.SC by random forest and support vector machine analysis, respectively. The biomarker panel, including the ratio of L-asparagine to L-aspartic acid, vanillylmandelic acid and glutaric acid, yielded an area under the receiver operating characteristic curve of 0.808.

**Conclusions:**

This study gives a holistic view of the metabolic phenotype of schizophrenia with violence which is characterized by the dysregulation of lipids and amino acids. These results might provide information for the aetiological understanding and management of violence in schizophrenia; however, this is a preliminary metabolomics study about schizophrenia with violence, which needs to be repeated in future studies.

## Background

Violence is a complex social behaviour and is frequently correlated with schizophrenia [1]. Although only a minority of schizophrenia patients are violent, this violence could lead to a large negative influence on the whole group of patients with schizophrenia. As a result, many studies aim to identify biomarkers of violence among schizophrenia patients to understand the cause of violence and to aid in the prediction and management of violence [2, 3].

In previous studies, the genes of monoamine oxidase A (MAOA) and catechol-O-methyltransferase (COMT) genes have been correlated with violence [4, 5]. Abnormalities in the amygdala [6, 7], limbic prefrontal cortex [8, 9] and hippocampus [10] have been repeatedly reported to be related to the violent behaviours. Although the neuroimaging findings of aggression and violence were heterogeneous, the structural and functional MRI results have consistently reported abnormalities in the orbitofrontal cortex (OFC) and anterior cingulate cortex (ACC) in schizophrenia [10]. In addition, many findings also suggest the dysfunctions of the cortico-limbic regions, involving the amygdala and prefrontal cortex, in schizophrenia with violence [3, 11]. In terms of neurotransmitters, the serotonergic system is one of the best studied areas of the neurobiology of violence. Studies have found that the relationship between serotonin (5-HT) and violence is complex, but these findings have suggested that 5-HT might influence violence through neural networks involving the amygdala, prefrontal cortex and the striatum [12]. These biomarkers have been applied to some violent behaviour prediction and legal practice [3], but they are hard to be change or regulate directly. It is necessary to explore new regulated biomarkers that could be beneficial to the targeted management of violence.

Metabolites are regulatable factors. Tryptophan is the amino acid precursor of serotonin (*5*-hydroxytryptamine, 5-HT) biosynthesis [13], and its dietary depletion can reduce central 5-HT concentration [14]. Studies have found that decreased plasma tryptophan levels are related to a higher rate of aggressive behaviours [15, 16]. Moreover, *Thurmond* reported higher tyrosine increased aggressive behaviours in animal experiments [17]. Earlier studies reported glycine mediates the defend reaction types in mouse behaviours [18]. In addition, low levels of cholesterol associated with aggression are frequently reported [19–22]. Recently, many studies have found dietary omega-3 supplementation can reduce violent behaviours in children, young men and schizophrenia patients [23–28].

Limited by techniques, many studies mainly focus on one or two metabolites; therefore, a holistic view of the metabolic phenotype of violence might provide more information to help us understand violence. Metabolomics is able to provide a holistic view. It has been applied to the diagnosis, mechanism of disease, the identification of new drug targets and the monitoring of treatment outcomes [29, 30]. To find diagnostic biomarkers of schizophrenia, the metabolomics studies were conducted in different samples, such as peripheral blood mononuclear cells [31], serum [32], plasma [33] and urine [34]. Furthermore, the signatures reported by metabolomics studies are in agreement with the polyunsaturated fatty acid metabolism hypothesis [35] and the membrane phospholipid hypothesis [36] of schizophrenia [37]. A study has validated the potential of tryptophan to be a biomarker for aggression [38]. Another study suggested using lipid levels to predict violent behaviours in schizophrenia [22]. Furthermore, untargeted metabolomics is a method that is independent of priori assumptions, which could provide a holistic approach to understanding the phenotype [39]. To our knowledge, studies have reported metabolome studies of violence in animal models [40–42], but none in schizophrenia patients. Consequently, we speculate that schizophrenia patients with violence (V.SC) might differ from schizophrenia patients without violence (NV.SC) in metabolic phenotype.

We hypothesized that V.SC would present metabolic characteristics that differ from those of NV.SC. Therefore, in this study, we compared the metabolic biomarkers of V.SC with those of NV.SC by using untargeted metabolomics and profiled the metabolic status of V.SC in a holistic perspective. This study also conducted a feature selection analysis to further explore the predictive utility of discovered metabolic biomarkers in discriminating schizophrenia patients with violence from those without violence.

## Methods

Patients were recruited from the inpatient department of Chengdu Compulsory Medical Center (25 samples) and Chengdu Jinxin Mental Health Center (52 samples), Sichuan, China. They were all aged 18–40 years, of Han nationality and lived in Sichuan in the last year. They were screened by psychiatric and forensic psychiatric graduate students using the Structured Clinical Interview for DSM-IV. Patients were ultimately diagnosed with schizophrenia by two senior psychiatrists according to DSM-IV criteria and the interview. They were naïve to using any antibiotics in the last 3 months. Patients were excluded if they had been diagnosed with hypertension, diabetes, cirrhosis, immunodeficiency, autoimmune diseases and cancer in the last 3 months. Patients with organic mental disorder, mental and behavioural disorders due to psychoactive substance use, mental retardation and alcohol/drug abuse or dependence were also excluded. Pregnant or breastfeeding women were excluded. Patients with a history of suicide/self-injury were excluded. The immediate families of all participants had signed the informed consent. The ethics approval of this study was approved by the Medical Ethics Committee of Sichuan University.

Patients were classified into two groups—schizophrenia patients with violence (V.SC) and schizophrenia patients without violence (NV.SC). Violent behaviour was identified according to the serious violence classification in the MacArthur Violence Risk Assessment Study (MVRAS): 1) batteries that resulted in physical injury or involved the use of a weapon, 2) sexual assaults, and 3) threats made with a weapon in hand [43]. If the patients acted any of the above behaviours since they were diagnosed with schizophrenia, they were assigned to the V.SC group. Patients who never acted any of the above behaviours were assigned to the NV.SC group.

### Psychiatric symptom assessment

A questionnaire was conducted to collect demographic information. The Positive and Negative Syndrome Scale (PANSS) was used by three psychiatric and forensic psychiatric graduate students to assess psychotic symptoms (ICC = 0.983).

### Untargeted metabolomics analysis

Fasting venous blood specimens were collected from 77 participants in EDTA anticoagulant tubes between 7:00 and 7:30 a.m.. The fresh blood samples were transported to the laboratory in cold chain (4 °C) in 20 min and then centrifuged at 1000 g, 4 °C to obtain plasma. The plasma was put in a liquid nitrogen tank for cold extraction for 15 min and then put in a − 80 °C freezer until analysis.

#### Plasma sample preparation for metabolomics

To separate the debris or lipid layer, thawed plasma samples were centrifuged at 3000 g, 4 °C for 5 min. For each sample, 50 μl of plasma was mixed with 10 μl of internal standard, and 175 μl of pre-cooled methanol/chloroform (v/v = 3/1) was added. The mixture was frozen at − 20 °C for 20 min and centrifuged at 14,000 g, 4 °C for 4 min. For all samples, the supernatant was concentrated to near dryness using a Centrivap vacuum concentrator (Labconco, Kansas City, MO, USA) to remove chloroform and was then further lyophilized using a freeze dryer (Labconco, Kansas City, MO, USA). Then, 50 μl of oximation reagent (a pyridine solution of methoxyamine hydrochloride, 20 mg/ml) was added to the dried sample, and incubated at 30 °C for 2 h. Then, 50 μl of silylation reagent (MSTFA+ with FAME) in 1% TMCS) was added to each sample, which was silanized for 1 h at 37.5 °C using an automatic injector. The derivatized sample was then injected into the GC-TOF/MS analyser using an automatic injector. The derivatization and injection of the above samples were performed by the MPS2 Multi-Function Autosampler.

#### GC-TOF/MS analysis

Each 1 μl derived sample was injected into gas chromatography time-of-flight mass spectrometer (GC-TOF/MS) system (Pegasus HT, Leco Corp., St. Joseph, MI, USA), with helium as the carrier gas at a flow rate of 1.0 ml/min. An Rxi-5 ms capillary column (30 m × 250 μm i.d., 0.25-μm film thickness; Restek corporation, Bellefonte, PA, USA) was used for metabolite separation. The injection temperature was set at 270 °C. The oven temperature programming was set as follows: maintaining 80 °C for 2 min, then raising to 300 °C at a rate of 12 °C/min, maintaining 300 °C for 4.5 min, then raising to 320 at a rate of 40 °C/min, and maintaining 320 °C for 1 min. The transfer interface and ion source temperature were set as 270 °C and 220 °C, respectively. The mass spectrometer adopted an electron impact ionization mode of − 70 eV, a detector voltage of − 1450 V, and an acquisition rate of 25 spectra/sec, in a full scan mode ranging from 50 to 550 amu.

#### Metabolomics data analysis

The retention index and mass spectrometry data were compared with the previous JiaLib™ metabolite database to complete the metabolite identification by using Xplore MET software (v3.0, Metabo-Profile, Shanghai, China) [44]. More details about the JiaLib™ metabolite database, Xplore MET software, raw mass spectral data processing and data preprocessing are provided in the *Supplement**.* Principal component analysis (PCA) was used to observe the trend of aggregation in group and the trend of separation between groups. Orthogonal partial least square discriminant analysis (OPLS-DA) were used to discriminate V.SC patients from NV.SC patients. PCA is an unsupervised modelling method that is commonly used to detect data outliers, clustering, and classification trends without prior knowledge of the sample set [45]. OPLS-DA has been widely used for multi-class classification and the identification of different changing metabolites [46, 47].

The Wilcoxon-Mann-Whitney rank sum test was used to select plasma metabolites that were significantly differed between V.SC and NV.SC (*P* > 0.05). Random forest (RF) analysis applies an ensemble technique by using bootstrap resampling technology and is an effective method for classification and feature selection [48, 49]. The support vector machine (SVM) is a machine learning classifier and can mitigate the effects of noisy data [50]. Both have been widely been employed for biomarker discovery [51, 52]. RF and SVM analyses were separately used to evaluate the importance of the differential plasma metabolites identified by the Mann-Whitney-Wilcoxon rank sum test and to select plasma metabolic biomarkers. RF analysis adopted Boruta algorithm (maxRun = 1000) by using Boruta package of R studio. Only if the *p* < 0.01, the feature can be identified as “confirmed” and the Bonferroni post-hoc correction was used in *p* value of results [48]. SVM analysis adopted 7 fold cross validation and repeated 100 times by using svm () function in R package e1071 [53]. Only the plasma metabolites that meet both RF (marked as “confirmed”) and SVM (importance > 60, or the number of metabolites with importance above 60 < 5, the five most important metabolites) feature selection criterion can be identified as metabolic biomarkers and selected to form final biomarker panel. Receiver operating characteristic (ROC) curve was used to assess the validity of plasma metabolic biomarkers in discriminate V.SC from NV.SC.

Metabolic pathway enrichment analysis (MPEA) is a commonly used method for metabolic pathway analysis. MPEA can help detect biologically meaningful metabolite sets that have been enriched in human metabolomic studies [54]. We used MPEA to identify differential metabolic pathways and illustrate meaningful metabolites in these ways.

## Results

### Clinical characteristics

The V.SC group consisted of 53 patients and the NV.SC group consisted of 24 patients. The duration of inpatients ranged from 0.2 to 14 years (V.SC: 0.3–14.0 years; NV.SC: 0.2–8.0 years) and differed significantly between groups. There was no statistically significant difference between the two groups in terms of other demographic characteristics and clinical symptoms. Risperidone and clozapine were the most frequently used antipsychotic drugs. In the V.SC group, 51 patients (96.2%) had batteries resulting in physical injury, 32 patients (60.4%) had batteries involving the use of a weapon, 9 patients (17.0%) made threats with a weapon in hand, and no patient had sexual assaults, after they were diagnosed with schizophrenia (Table 1).

**Table 1:** Comparison of the demographic characteristics, clinical symptoms and violence situation between the V.SC and NV.SC groups

	NV.SC *N* = 24		V.SC *N* = 53		t/χ^2^	*P*
Age (Mean, SD)	30.9	5.2	32.6	4.7	−1.45	0.15
Male (*N*, %)	13	54.2	38	71.7	2.27	0.13
Marriage status (*N*, %)						
Married	6	25.0	6	11.3	2.91	0.23
Divorce	2	8.3	9	17.0		
Alone	16	66.7	38	771.7		
BMI (Mean, SD)	23.0	4.8	22.8	3.0	0.13	0.90
Educational years (Mean, SD)	10.3	4.4	9.7	3.9	0.60	0.55
Occupation(*N*, %)						
Other jobs	5	20.8	5	9.4	1.98	0.37
Peasantry	2	8.3	4	7.5		
Unemployed	17	70.8	44	83.0		
Smoking (*N*, %)	10	41.7	29	54.7	1.13	0.29
First-episode SC. (*N*, %)	3	12.5	2	3.8	2.08	0.15
Duration of SC. (Mean, SD, year)	9.1	4.0	9.5	5.4	−0.34	0.74
Duration of inpatient (Mean, SD, year)	1.7	2.3	3.7	3.7	−2.41	0.02
Duration of antipsychotic treatment (Mean, SD, year)	8.0	4.7	8.6	5.4	−0.46	0.64
Risperidone (*N*, %)	11	45.8	23	43.4	0.04	0.84
Clozapine (*N*, %)	10	41.7	30	56.6	1.48	0.22
Violent behaviours during SZ. (*N*, %)						
Batteries resulting in physical injury	0	0	51	96.2	68.40	0.00
Batteries involving the use of a weapon	0	0	32	60.4	24.80	0.00
Sexual assaults	0	0	0	0	/	/
Threats made with a weapon in hand	0	0	9	17.0	4.62	0.03
PANSS (Mean, SD)						
Positive symptom	11.5	7.0	10.7	4.8	0.61	0.54
Negative symptom	16.0	8.6	15.7	8.0	0.17	0.87
General psychotic symptom	27.2	9.0	24.9	5.3	1.42	0.16
PANSS total score	54.8	19.7	50.8	12.6	1.04	0.30

Abbreviation: *SC* schizophrenia, *V.SC* schizophrenia patients with violence, *NV.SC* schizophrenia patients without violence

### Plasma metabolic profiles of schizophrenia with violence

A total of 236 plasma metabolites were detected by GC-TOF/MS analysis, among which 129 were annotated by JiaLib™ metabolite database.

#### Discrimination model

**Fig. 1:**
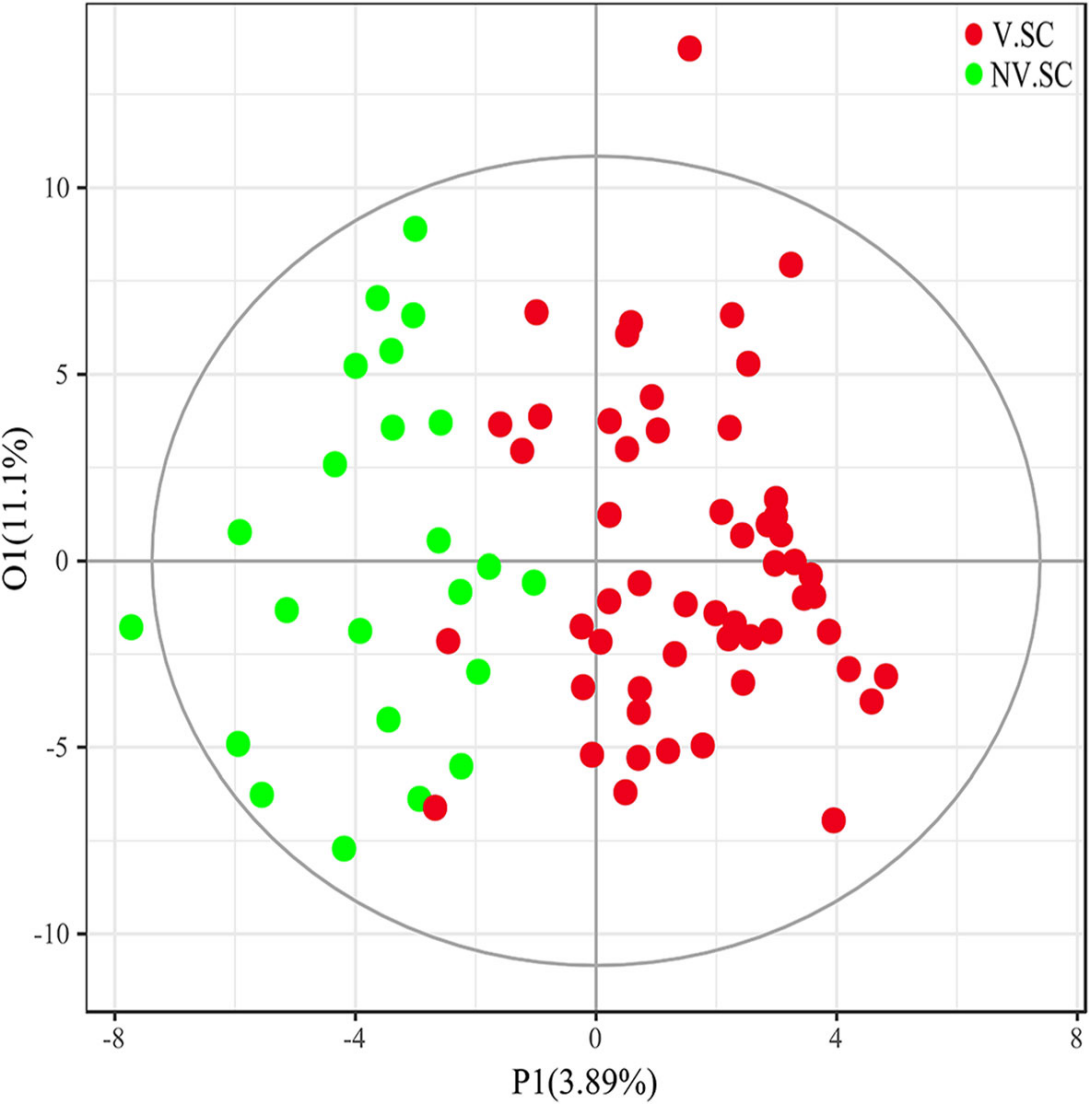
Orthogonal partial least square discriminant analysis (OPLS-DA) was used to discriminate the V.SC group from the NV.SC group. V.SC: schizophrenia patients with violence; NV.SC: schizophrenia patients without violence

The PCA shows no separation trend between the V.SC and NV.SC groups (Supple. Figure 1 in *Supplement*). The OPLS-DA model shows poor predictive utility in discriminating V.SC group and NV.SC group (R^2^Y = 0.669, Q^2^ = 0.112) (Fig. 1).

#### Group differences

**Fig. 2:**
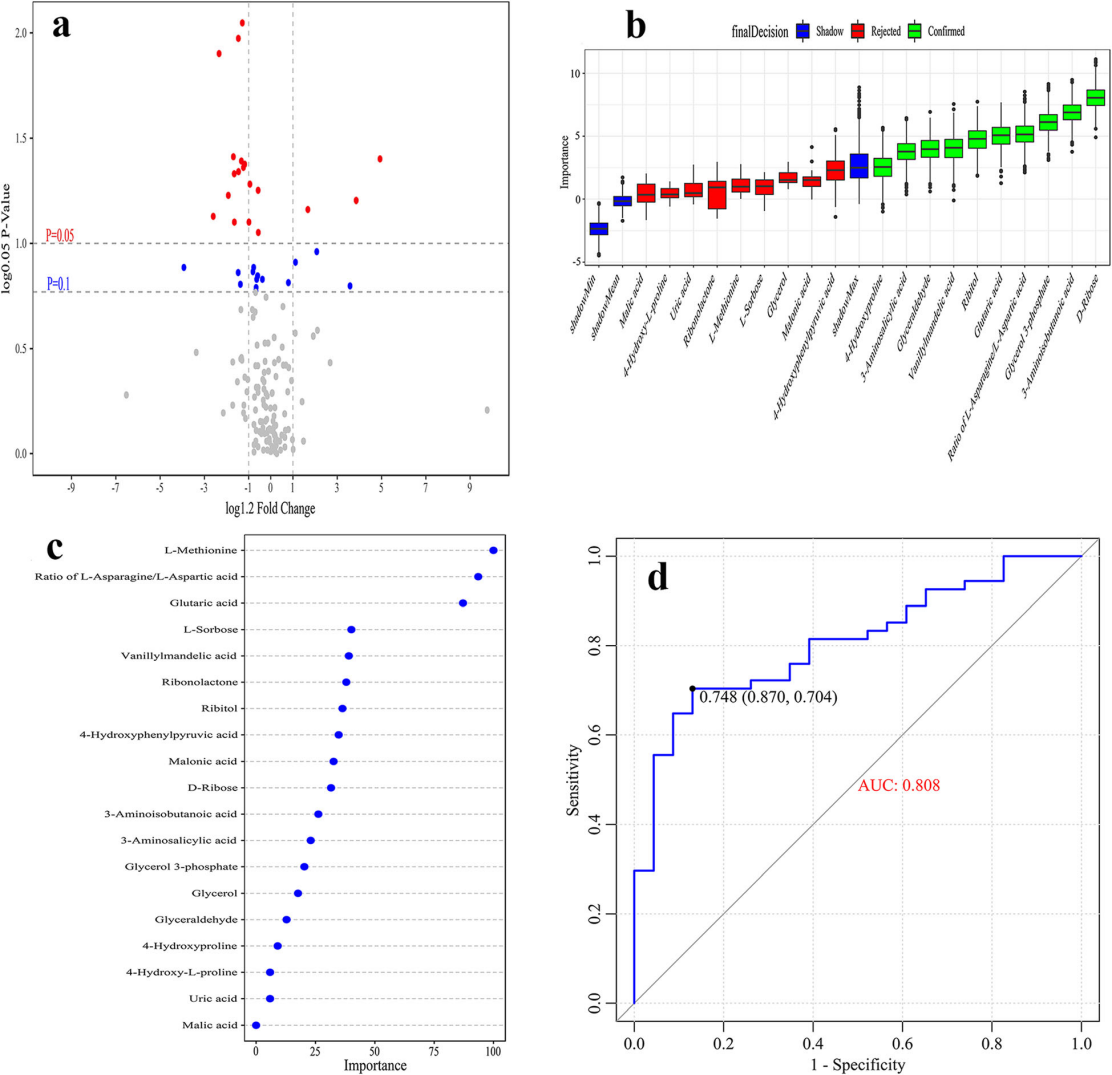
Metabolic biomarkers of V.SC. **a** The enhanced volcano plot shows the differential plasma metabolites between the V.SC group and the NV.SC group. **b** Random forest (RF) analysis assessed the feature importance of the differential plasma metabolites. **c** Support vector machine (SVM) analysis assessed the feature importance of the differential plasma metabolites. **d** Receiver operating characteristic (ROC) curve analysis assessed the utility of a metabolic biomarker panel in discriminating V.SC from NV.SC. V.SC: schizophrenia patients with violence; NV.SC: schizophrenia patients without violence

In univariate analysis, nineteen plasma metabolites differed significantly between the V.SC group and NV.SC group (*P* < 0.05), including seven organic acids, five amino acids, four carbohydrates, one lipid, one aldehyde and one alcohol. Three metabolites increased in V.SC, and their fold change (FC) of them ranged above 1.2. Sixteen metabolites decreased in V.SC, and the FC of twelve metabolites ranged below 0.83. The nineteen differential metabolites mainly participated in amino acid metabolism, lipid metabolism and carbohydrate metabolism (Fig. 2a and Table 2).

**Table 2:** Differential plasma metabolites selected by Wilcoxon-Mann-Whitney rank sum test between the V.SC and NV.SC groups

Pathway	Metabolites	FC	*P* value
Amino acid metabolism	Vanillylmandelic acid	2.46	0.02
	Malic acid	0.90	0.04
	4-Hydroxyphenylpyruvic acid	0.80	0.02
	4-Hydroxy-L-proline	0.84	0.04
	L-Methionine	0.79	0.002
	Ratio of L-asparagine/L-aspartic acid	0.77	0.003
Lipid metabolism	Malonic acid	0.77	0.02
	Glycerol	2.02	0.03
	Glycerol 3-phosphate	0.71	0.03
	Glyceraldehyde	1.36	0.03
Pentose phosphate pathway	D-Ribose	0.74	0.02
Purine metabolism	Uric acid	0.74	0.04
Pyrimidine metabolism	3-Aminoisobutanoic acid	0.84	0.02
NA	L-Sorbose	0.74	0.02
	3-Aminosalicylic acid	0.90	0.02
	Glutaric acid	0.65	0.00
	4-Hydroxyproline	0.62	0.03
	Ribitol	0.81	0.02
	Ribonolactone	0.79	0.02

Abbreviation: *FC* fold change, *NA* not available, *V.SC* schizophrenia patients with violence, *NV.SC* schizophrenia patients without violence

### Biomarker panel selection

#### Assessment of importance

We used RF analysis to assess the importance (Imp) of the nineteen differential metabolites and nine metabolites were excluded. Ten metabolites were confirmed as metabolic biomarkers of the V.SC group by RF analysis (Imp> 2.5) (Fig. 2b), including D-ribose, 3-aminoisobutanoic acid, glycerol 3-phosphate, ratio of L-asparagine to L-aspartic acid, glutaric acid, ribitol, vanillylmandelic acid, glyceraldehyde, 3-aminosalicylic acid and 4-hydroxyproline.

We also used RF analysis to assess the importance of the nineteen differential metabolites. We selected the five most important metabolites as metabolic biomarkers of the V.SC group, including L-methionine, the ratio of L-asparagine to L-aspartic acid, glutaric acid, L-sorbose and vanillylmandelic acid (Fig. 2c).

To avoid over-fitting issue, only the plasma metabolites that meet both RF and SVM feature selection criterion can be identified as metabolic biomarkers and selected to form final biomarker panel to discriminate the V.SC group from the NV.SC group. Three plasma metabolites meet the criterion, including ratio of L-asparagine to L-aspartic acid, vanillylmandelic acid and glutaric acid. The biomarker panel formed by the three metabolites, yielded an area under the receiver operating characteristic curve (AUC) of 0.808 (Fig. 2d).

### Differential metabolic pathways

**Fig. 3:**
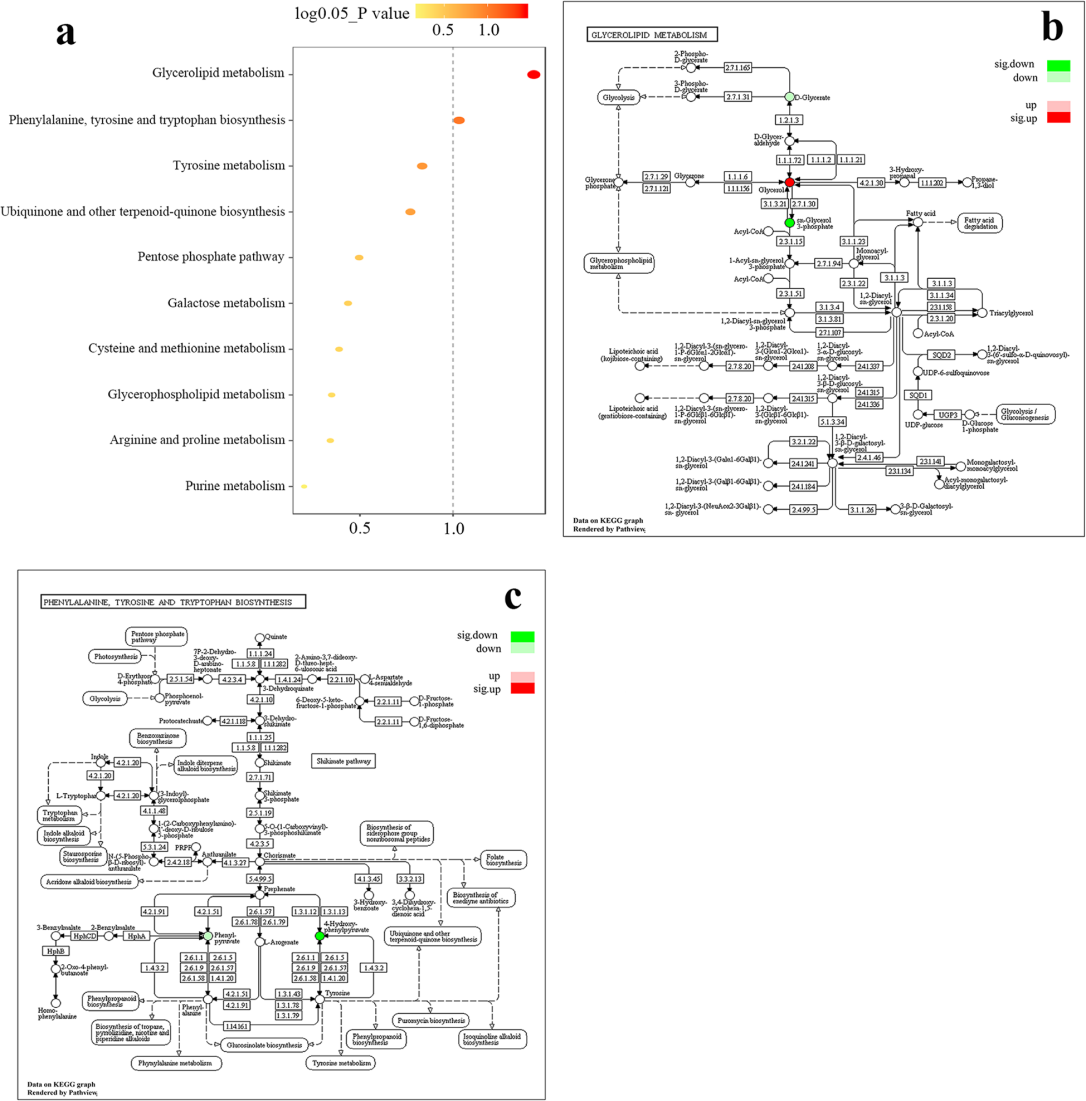
Metabolic pathways between the V.SC and NV.SC groups. **a** Metabolic pathways are presented according to the *P* value calculated by metabolic pathway enrichment analysis (MPEA). **b** The metabolite regulatory network of the glycerolipid metabolism pathway. **c** The metabolite regulatory network of the phenylalanine, tyrosine and tryptophan biosynthesis pathway. V.SC: schizophrenia patients with violence; NV.SC: schizophrenia patients without violence

Two metabolic pathways, glycerolipid metabolism and phenylalanine, tyrosine and tryptophan biosynthesis were identified as significantly differential pathways between the V.SC and NV.SC groups by MPEA (Fig. 3a and Table 3). In the glycerolipid metabolism pathway, glycerol was significantly up-regulated and glycerol 3-phosphate was significantly down-regulated (Fig. 3b). In the phenylalanine, tyrosine and tryptophan biosynthesis pathway, 4-hydroxyphenylpyruvic acid was significantly down-regulated (Fig. 3c).

**Table 3:** Differential metabolic pathways identified by MPEA between the V.SC and NV.SC groups

Pathway	Up	Down	p_hyper
Glycerolipid metabolism	Glycerol	Glycerol 3-phosphate	0.01
Phenylalanine, tyrosine and tryptophan biosynthesis		4-Hydroxyphenylpyruvic acid	0.045
Tyrosine metabolism	Vanillylmandelic acid	4-Hydroxyphenylpyruvic acid	0.08
Ubiquinone and other terpenoid-quinone biosynthesis		4-Hydroxyphenylpyruvic acid	0.10
Pentose phosphate pathway		D-Ribose	0.23
Galactose metabolism	Glycerol		0.27
Cysteine and methionine metabolism		L-Methionine	0.31
Glycerophospholipid metabolism		Glycerol 3-phosphate	0.35
Arginine and proline metabolism		4-Hydroxyproline	0.36
Purine metabolism		Uric acid	0.55

Abbreviation: *MPEA* metabolic pathway enrichment analysis, *V.SC* schizophrenia patients with violence, *NV.SC* schizophrenia patients without violence

## Discussion

After controlling for confounding factors, we profiled metabolic status and identified 19 differential metabolites in the plasma of V.SC by comparing with NV.SC. The biomarker panel, including the ratio of L-asparagine to L-aspartic acid, vanillylmandelic acid and glutaric acid, achieved a good classifier for discriminating schizophrenia patients with violence (AUC = 0.808).This study detected metabolite status in schizophrenia patients with violence and found metabolic biomarkers of schizophrenia with violence. Our results can not only aid in understanding the aetiology of violence, but also suggest the plasma metabolites could serve as a new biomarkers for predicting violence among schizophrenia patients.

Our results showed glycerolipid metabolism and related metabolites (including glycerol, glycerol 3-phosphate and glyceraldehyde) differed between V.SC and NV.SC. Notably, the patients of this study, being hospital inpatients, share similar diets, living conditions and living habits, which are confounding factors of metabolites [55–57]. Although the duration of inpatient showed difference between groups, the average years of each group was above one year. This means they had shared similar environment for an enough long time, so we do not think this could contribute much to the difference of metabolites. Glycerol 3-phosphate is a phosphoric ester of glycerol that controls the flux through the glycerolipid/free fatty acid cycle, phospholipid biosynthesis and many other cellular functions [58]. We found the glycerol 3-phosphate was decreased in V.SC, which suggests a dysregulation of lipid metabolism in schizophrenia patients with violence. This founding is consistent with previous studies reporting the lower fatty acid levels might be related to violence [23, 59]. Although earlier studies have reported the serum cholesterol, a subclass of lipid [60], is uncorrelated with violence [61, 62], more recent studies have consistently found the increased violence risk is correlated with low cholesterol concentration [19, 20, 63] and polyunsaturated fatty acid [26, 59]. Omega-3 supplement can reduce violent behaviours in children [25], young men [64], schizophrenia patients [26] and adult prisoners [23]. Our research also proves the important role of lipid metabolism in regulating violence.

In this study, five amino acids decreased in V.SC compared with NV.SC, involving in nine amino acid metabolism pathways; phenylalanine, tyrosine and tryptophan biosynthesis was a differential metabolic pathway between the two groups. The dysregulation of amino acids has been consistently correlated with psychopathology [65, 66]. Some amino acids are correlated with violence in previous studies, especially tryptophan [67], which is the amino acid precursor of 5-HT biosynthesis [13]. A study of violent criminals found the plasma content of the amino acid tryptophan and other large neutral amino acids were increased [67–69]; however, much more studies have reported decreased tryptophan is correlated with aggressive behaviours [15, 70–72]. Instead of finding the plasma tryptophan as a differential metabolite, we found phenylalanine, tyrosine and tryptophan biosynthesis was a differential metabolic pathway between the schizophrenia patients with and without violence, which support amino acids play an important role in regulating violence.. We found vanillylmandelic acid, involved in tyrosine metabolism, was increased in V.SC, which is consistent with an earlier study reporting tyrosine supplements increased aggressive behaviours in mice [17]. Limited by techniques, many previous studies reported one or several amino acids were altered among individuals with violence, based on one or another hypothesis. Our results give a holistic view of the metabolic phenotype of violence in schizophrenia. Metabolomics should be further used to explore the role of amino acids in violence in the future.

We identified L-methionine, the ratio of L-asparagine to L-aspartic acid, glutaric acid, L-sorbose and vanillylmandelic acid as predictors of violence in schizophrenia patients. A previous study reported serotonin/tryptophan *1000, antisocial behaviour and global assessment of functioning were good predictors of aggressive behaviours among inmate (AUC = 0.851) [38]. Metabolic predictors always showed variability in different studies with schizophrenia patients [32, 73, 74]; however, some consistent potential biomarkers of schizophrenia have been discovered [37]. Consequently, more metabolomics studies should be conducted to discover metabolic biomarkers of violent behaviours in schizophrenia.

There are also some limitations in this study. First, the sample size was not 1:1 between the two groups. This was led by controlling confounding factors, such as diet, disease and so on. Second, the sample size was small and there can be over-fitting issue in classification. Small sample might be the reason of why the orthogonal partial least square discriminant analysis failed to distinguish V.SC from NV.SC. Because of controlling confounding factors of metabolites, it is difficult to collect other sample to expand sample size or validate over-fitting. We used two different ways (RF and SVM) to select metabolic biomarkers from differential metabolites and chosen overlapping part of the two results (RF and SVM) to form final biomarker panel. This could not only remedy the disadvantage of small sample, but also reduce the over-fitting issue in some degree, for it might be unlikely for two different algorithms to over-fitting the same way [48, 75]. The result of ROC analysis also validate the utility of the final biomarker panel. However, this is a preliminary metabolomics study about schizophrenia with violence, our biomarker panel of schizophrenia with violence needs further validation in an independent sample of violent schizophrenia. Third, the length of inpatients duration is relative long. However, the minimum interval (about two months) could be enough long for the corresponding changes of plasma metabolites. Finally, the fresh blood samples were not collected immediately after the patients conducted violent behaviour, therefore the violence in this study is more inclined to be a feature than a status.

## Conclusions

Our study gives a holistic view of the metabolic phenotype of violence in schizophrenia by untargeted metabolomics. We found the dysregulation of lipids and amino acids featured the metabolic phenotype of schizophrenia with violence. These results further confirm violent behaviours might be correlated with a dysregulated metabolic status, suggest that severe metabolic dysregulation might be related to violence and provide information for the aetiological understanding and management of violence in schizophrenia in the future.

## Supplementary information

**Additional file 1.** Supplementary methods and materials. **Supple. Table 1.** Receiver Operating Characteristic (ROC) results of differential metabolites. **Supple. Figure 1.** Principal components analysis (PCA) of the V.SC group and the NV.SC group.

## Data Availability

The datasets generated and/or analysed during the current study are not publicly available since the publication of raw data was not included in the consent forms, but all data and materials are available from the corresponding author on reasonable request.

## Abbreviations

AUC: Area under curve
FC: Fold change
MPEA: Metabolic pathway enrichment analysis
NV.SC: Schizophrenia patients without violence
PANSS: Positive and Negative Syndrome Scale
RF: Random forest
ROC: Receiver operating characteristic curve
SVM: Support vector machine
V.SC: Schizophrenia patients with violence
