# Supplementary material for: Dysregulation of amino acids and lipids metabolism in schizophrenia with violence
Source: BMC Psychiatry. 2020 Mar 4;20:97. doi: 10.1186/s12888-020-02499-y (PMC7055102; doi:10.1186/s12888-020-02499-y)
Supplement: Supplementary file 1 — Additional file 1. Supplementary methods and materials. Supple. Table 1. Receiver Operating Characteristic (ROC) results of differential metabolites. Supple. Figure 1. Principal components analysis (PCA) of the V.SC group and the NV.SC group. [file 12888_2020_2499_MOESM1_ESM.docx]

**Supplementary methods and materials**

Jialib metabolite database

The current JiaLib comprises over 1,200 mammalian metabolites with 15-year accumulation, which is one of the most comprehensive metabolite libraries in the world. The reference chemicals presented in JiaLib were commercially purchased from Sigma-Aldrich (St. Louis, MO, USA), Santa Cruz (Dallas, TX, USA), Nu-Chek Prep (Elysian, MN, USA), and synthesized in the laboratory.

XploreMET software

XploreMET software (v3.0, Metabo-Profile, Shanghai, China) is a powerful 1-STOP solution for GC-MS-based metabolomics through more than a decade of research and development. The software integrates one of the most extensive metabolite database-JiaLib in the world and streamlines procedures for raw data processing, peak deconvulation, compound annotation, statistical analysis, pathway analysis, and project report within minutes of completing the analytical sequence. The current XploreMET is hosted on Dell PowerEdge R730 Servers operated with Linux Ubuntu 16.10 OS. The secured Java UI (User Interface) permits the users have access to the raw data and a great variety of statistical tools for viewing and exploring project data with their own desire.

Raw Mass Spectral Data Processing

The raw data generated by GC-TOF/MS were processed using XploreMET for automated baseline denosing and smoothing, peak picking and deconvultion, creating reference database from the pooled QC samples, metabolite signal alignment, missing value correction and imputation, and QC correction.

Data Preprocessing

Each data set was transformed into comparable data vectors for statistical analysis. All measurements were mean-centered and scaled by the standard deviation of the observed measurements.

Parameters for machine learning methods

Random Forest (RF)

RF analysis adopted Boruta algorithm (maxRun = 1000) by using Boruta package of R studio.

Confirmed: p<0.01

Max interations: 1000

mcAdj = TURE，Bonferroni post-hoc correction was used in p value of results.

Support vector machine (SVM)

Kernel function: the kernel uses radial basis kernel (rbf kernel, default, as shown below).


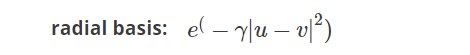


gamma: it's the kernel coefficient of 'rbf', 'poly' and 'sigmoid'. We set gamma to 'auto' with a value of 1 / N (n is the characteristic number).

Cost: 'C' - constant regularization coefficient (penalty parameter): 1

tolerance of termination criterion (default: 0.001)

use shrinking heuristic method or not: we chose “yes”, use this method.

epsilon in the insensitive-loss function：0.5

If there are no special instructions, SVM and boruta methods use the default value in R.

**Supplementary Results**

**Supple. Table 1** Receiver Operating Characteristic (ROC) results of differential metabolites.

| **Differential Metabolites** | **AUC** | **95% C.I.** | **Specificity** | **Sensitivity** |
| --- | --- | --- | --- | --- |
| L-Methionine | 0.72 | 0.60-0.84 | 0.78 | 0.59 |
| Ratio of L-Asparagine/L-Aspartic acid | 0.72 | 0.59-0.842 | 0.65 | 0.70 |
| Glutaric acid | 0.71 | 0.58-0.85 | 0.78 | 0.72 |
| L-Sorbose | 0.68 | 0.55-0.81 | 0.78 | 0.57 |
| Vanillylmandelic acid | 0.68 | 0.54-0.82 | 0.61 | 0.76 |
| Ribonolactone | 0.68 | 0.54-0.81 | 0.70 | 0.67 |
| Ribitol | 0.67 | 0.53-0.82 | 0.57 | 0.87 |
| 4-Hydroxyphenylpyruvic acid | 0.67 | 0.53-0.82 | 0.61 | 0.76 |
| Malonic acid | 0.67 | 0.53-0.81 | 0.65 | 0.69 |
| D-Ribose | 0.67 | 0.52-0.82 | 0.48 | 0.94 |
| 3-Aminoisobutanoic acid | 0.67 | 0.52-0.81 | 0.35 | 1.00 |
| 3-Aminosalicylic acid | 0.66 | 0.54-0.79 | 0.87 | 0.56 |
| Glycerol 3-phosphate | 0.66 | 0.50-0.83 | 0.65 | 0.80 |
| Glycerol | 0.66 | 0.53-0.79 | 0.74 | 0.63 |
| Glyceraldehyde | 0.66 | 0.52-0.80 | 0.74 | 0.61 |
| 4-Hydroxyproline | 0.65 | 0.50-0.81 | 0.57 | 0.80 |
| 4-Hydroxy-L-proline | 0.65 | 0.51-0.79 | 0.61 | 0.72 |
| Uric acid | 0.65 | 0.50-0.80 | 0.61 | 0.74 |
| Malic acid | 0.65 | 0.51-0.79 | 0.61 | 0.69 |

**Abbreviation:** AUC, area under curve.

**Supple. Figure 1** Principal components analysis (PCA) of the V.SC group and the NV.SC group.


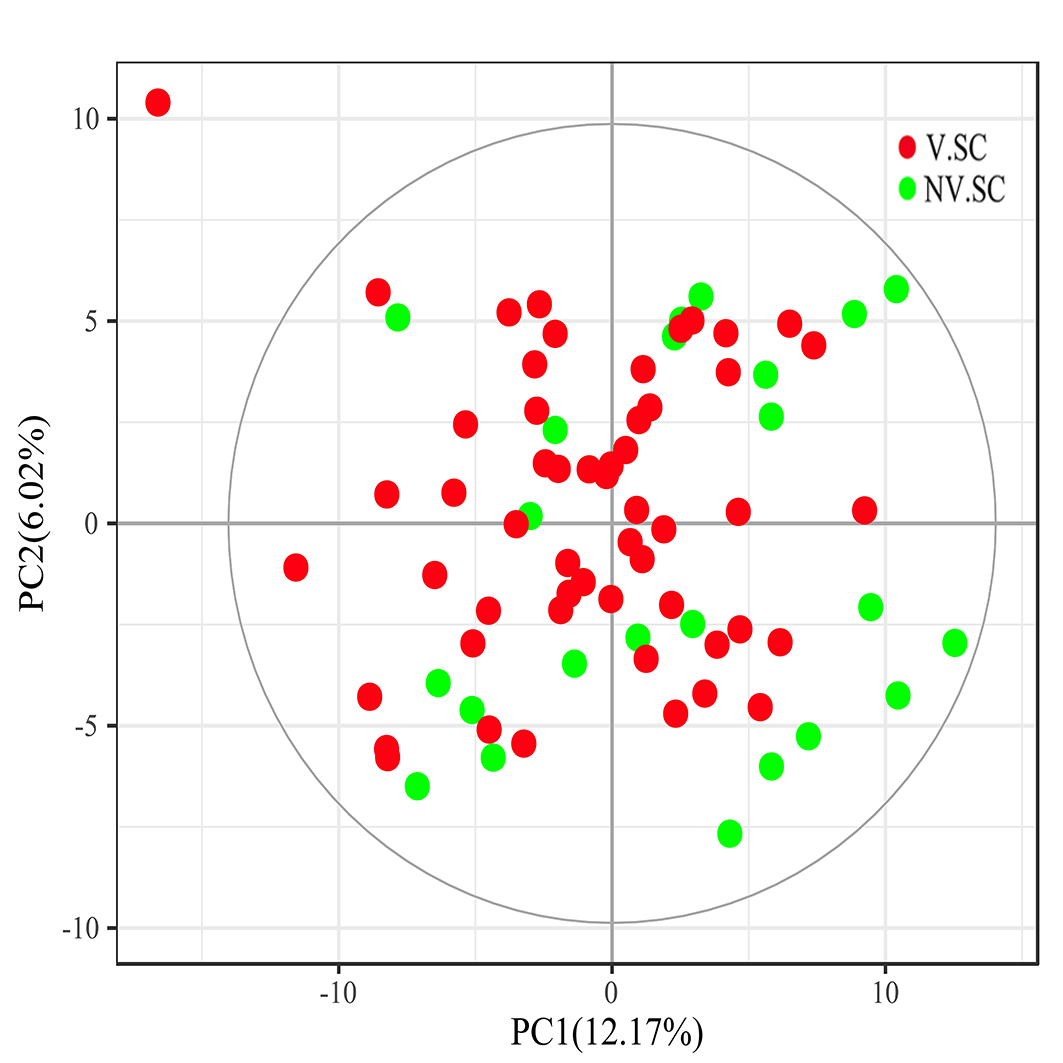


**Abbreviation:** V.SC: schizophrenia patients with violence; NV.SC: schizophrenia patients without violence.
